# Performance of model-based multifactor dimensionality reduction methods for epistasis detection by controlling population structure

**DOI:** 10.1186/s13040-021-00247-w

**Published:** 2021-02-19

**Authors:** Fentaw Abegaz, François Van Lishout, Jestinah M. Mahachie John, Kridsadakorn Chiachoompu, Archana Bhardwaj, Diane Duroux, Elena S. Gusareva, Zhi Wei, Hakon Hakonarson, Kristel Van Steen

**Affiliations:** 1 GIGA-R, Medical Genomics – BIO3, University of Liège, Liège, Belgium; 2 Department of Computer Science, New Jersey Institute of Technology, Newark, NJ USA; 3 Center for Applied Genomics, The Children’s Hospital of Philadelphia, Philadelphia, PA USA; 4 Department of Pediatrics, Division of Human Genetics, The Perelman School of Medicine, University of Pennsylvania, Philadelphia, PA USA; 5 WELBIO (Walloon Excellence in Lifesciences and Biotechnology), University of Liège, Liège, Belgium

**Keywords:** Epistasis, Population structure, Confounding, GWAS, GWAIS, MB-MDR, Gene-gene interaction, Population stratification, Principal components

## Abstract

**Background:**

In genome-wide association studies the extent and impact of confounding due to population structure have been well recognized. Inadequate handling of such confounding is likely to lead to spurious associations, hampering replication, and the identification of causal variants. Several strategies have been developed for protecting associations against confounding, the most popular one is based on Principal Component Analysis. In contrast, the extent and impact of confounding due to population structure in gene-gene interaction association epistasis studies are much less investigated and understood. In particular, the role of nonlinear genetic population substructure in epistasis detection is largely under-investigated, especially outside a regression framework.

**Methods:**

To identify causal variants in synergy, to improve interpretability and replicability of epistasis results, we introduce three strategies based on a model-based multifactor dimensionality reduction approach for structured populations, namely MBMDR-PC, MBMDR-PG, and MBMDR-GC.

**Results:**

Simulation results comparing the performance of various approaches show that in the presence of population structure MBMDR-PC and MBMDR-PG consistently better control type I error rate at the nominal level than MBMDR-GC. Moreover, our proposed three methods of population structure correction outperform MDR-SP in terms of statistical power.

**Conclusion:**

We demonstrate through extensive simulation studies the effect of various degrees of genetic population structure and relatedness on epistasis detection and propose appropriate remedial measures based on linear and nonlinear sample genetic similarity.

**Supplementary Information:**

The online version contains supplementary material available at 10.1186/s13040-021-00247-w.

## Background

Genome-Wide Association Studies (GWAS) are an effective approach for identifying genetic variants associated with disease risk [1]. In the context of such studies, population stratification refers to systematic ancestry differences between cases and controls [1]. The phenomenon is of particular concern in study designs with unrelated individuals. In contrast, family-based genetic association studies offer protection from population stratification, by using family data as internal controls, although at the expense of some loss of power from genotypic overmatching [2, 3]. For case-control genetic association studies, spurious associations are caused by the co-occurrence of two factors: a difference in the proportion of individuals from two (or more) subpopulations in cases and controls, and subpopulations having differing allele frequencies at the locus under investigation. This is in fact a special case of Simpson’s Paradox [4]. In general, this statistical phenomenon causes a potential bias in data analysis and occurs when a relationship or association between two variables reverses when a third factor, called a confounding variable, is introduced. The paradox also occurs if an association reverses when the data are aggregated over a third variable. Increasing the sample size is usually not a remedy for this issue, but may worsen the problem [5]. Several causes exist for population stratification. The basic one being shared genetic ancestry as a result of non-random mating between subgroups in a population due to various reasons, which may include social, cultural, or geographical ones. From an evolutionary point of view, not only population stratification but also admixture (i.e., inter-mating between genetically distinct groups) is created by human mating patterns. Potential consequences of population stratification are confounding, cryptic relatedness (i.e., unobserved ancestral relationships between individual cases and controls causing them to be non-independent), and selection bias [6, 7].

In case/control GWA studies, several strategies have been introduced in the literature for protecting against population structure mainly based on Principal Components Analysis (PCA). In contrast, the extent and impact of confounding due to population structure in gene-gene interaction studies are much less investigated and understood. However, the growing interest in the importance of detecting gene-gene interactions in the development and progression of complex diseases has led to the development of several tools; to name but a few: generalized linear regression models (GLM), BOOST [8], Model-Based Multifactor Dimensionality Reduction (MB-MDR) [9, 10], Multifactor Dimensionality Reduction (MDR) [11], Random Forest [12], PLINK [13], BiForce [14], Bayesian Models (e.g., BEAM) [15] and several others. For extensive reviews and appropriate references, please refer to [16–20]. However, the literature on epistasis detection in structured populations is very limited, apart from scenarios using a regression framework for association testing. On the other hand, Model-Based Multifactor Dimensionality Reduction (MB-MDR) offers a general framework and software tool for epistasis detection that can offer flexible maneuvering between different measurement scales for phenotypes and genomic predictors [9, 10, 21]. The MDR-SP method [22] combines MDR [11] with ideas implemented in the EIGENSTRAT software [23], a widely used software in GWAS that detects and corrects for population stratification via PCA.

In this article, we introduce strategies to account for population structure in epistasis studies using the MB-MDR framework. In particular, for the remainder of this article, we restrict attention to case-control study designs (binary original traits) and biallelic Single Nucleotide Polymorphisms (SNPs) as genetic markers. We propose and fully describe three strategies: i) MBMDR-PC, ii) MBMDR-PG and iii) MBMDR-GC. In MBMDR-PC, principal components (PCs) adjusted phenotypes but original genotypes are used to detect epistatic SNP pairs, similar to [23]. In MBMDR-PG adjusted phenotypes are obtained from fitting logistic mixed (polygenic) models on the original binary trait, hereby allowing to adjust for additional structures such as those arising from family relationships and cryptic relatedness. In MBMDR-GC, we follow principles of Genomic Control correction in GWAS but allow for multi-locus adaptivity. These methods are evaluated via extensive simulation studies which, to our knowledge, are unique in that complex nonlinear population structures, in the form of structural epistasis, are considered as well. Here, we let structural epistasis refer to the presence of interacting markers driving population differences or population substructure. All proposed strategies are formally compared to MDR-SP [22] in terms of type I error control and statistical power. Our work is important as it highlights the impact of nonlinear genetic population substructure on epistasis signal detection in GWAIS (Genome-Wide Association Interaction Studies).

## Material and methods

All proposed genome-wide epistasis screening strategies in structured populations are built on the Model-Based Multifactor Dimensionality Reduction (MB-MDR) method [10, 24, 25], as implemented in version 4.4.1. Detailed descriptions are provided in the aforementioned references. In a nutshell, MB-MDR was developed as a response to MDR [11] to address the following important points in an epistasis analysis, including 1) the need to correct for lower-order effects and to adopt flexible null hypotheses (no genetic effects whatsoever or no epistasis effects but possibly lower-order effects); 2) the acknowledgment of multi-locus genotype combinations with little power or no evidence towards increased or decreased disease risk; 3) the implementation of dimensionality reduction based on contrast testing of multilocus genotype combinations instead of testing each multilocus genotype combination against a pre-specified threshold, boosting performance in the presence of genetic heterogeneity. Even though the MB-MDR framework can be used for higher-level interaction detection and various outcome measurement scales and study designs, here we restrict attention to pair-wise interactions with default settings, including lower-order genetic effects correction and multiple testing correction via MaxT [10], unless specified otherwise. We describe the newly introduced methods as follows.

### MBMDR-PC: accounting for genomic structure by PCs

In MBMDR-PC, similar to EIGENSTRAT [23], we use either linear or nonlinear (kernel) PCs to correct for population structure. The popular EIGENSTRAT software to correct for population structure in GWAS contexts uses top linear PCs as covariates in a multiple regression [23]. It is a common practice to take between 2 and 10 principal components for correcting population structure in GWAS involving several countries. Many ad hoc procedures and formal statistical tests exist to determine the optimal number of principal components to correct for population structure [26] . Even though linear PCA is most popular and adequate in most cases to capture ancestry genetic background, PCA may fail to capture nonlinear population structure in genetics as shown in [27]. The nonlinear method developed by Alanis-Lobato and colleagues is based on a non-centered Minimum Curvilinear Embedding (ncMCE) kernel. Whereas the latter can better capture phylogenetic signals in samples, PCA better seems to reflect geographic dependencies [28]. Alternatively, kernel-based PCA can be adopted to account for nonlinear structures in high dimensional genetics data. In case-control epistasis studies, where the phenotype *Y* represents disease status (1 affected, 0 unaffected), the newly adjusted phenotype ${Y}_i^{adj}$ can be computed by fitting a logistic regression using the first few (linear or nonlinear) principal components (*W*_1_, ⋯, *W*_*r*_) and subtracting model-fitted values from observed phenotype values:

$$ \mathrm{logit}\left({\pi}_i\right)=\alpha +{\beta}_1{W}_{i1}+\cdots +{\beta}_r{W}_{ir}, $$

$$ {Y}_i^{adj}={Y}_i-{\hat{\pi}}_i, $$

$$ \mathrm{where}\ {\hat{\pi}}_i=\frac{\exp \left(\hat{\alpha}+{\hat{\beta}}_1{W}_{i1}+\cdots +{\hat{\beta}}_r{W}_{ir}\right)}{1+\exp \left(\hat{\alpha}+{\hat{\beta}}_1{W}_{i1}+\cdots +{\hat{\beta}}_r{W}_{ir}\right)}. $$

The newly adjusted phenotype *Y*^*adj*^ is taken as input to classic MB-MDR, in an attempt to capture genetic interactions that are not spurious due to inadequate handling of population structures. Detail of the MBMDR-PC approach is outlined in [29, 30].

### MBMDR-PG: accounting for genomic structure due to families and cryptic relatedness via the extended polygenic model

Family structure or cryptic relatedness may induce phenotypic similarity between individuals and may confound gene-phenotype associations in GWAS when not properly accounted for. Whereas PCs have proven useful in GWAS and structured populations due to shared genetic ancestry, they are not suitable to adequately protect for the effects of familial or cryptic relatedness on GWAS [1]. With the recent developments of computationally efficient algorithms, mixed models have become feasible in the context of GWAS as well as GWAIS, in structured populations, whether this structure presents population stratification, known or unknown relatedness. For quite some time, GWAS for binary traits have been analyzed with linear mixed models, assuming that little harm is done when sample sizes are in the thousands as is often the case with consortium data [31]. However, Chen et al. [32] showed that linear mixed models are inappropriate for analyzing binary traits when population stratification induces violation of the constant residual variance assumption in linear mixed models. Therefore, these authors developed a computationally efficient logistic mixed model for binary trait GWAS in the presence of population structure as well as familial and cryptic relatedness. In the same spirit of the logistic regression models adopted before, a logistic mixed model that includes interaction effect between two SNPs can be defined as

$$ \mathrm{logit}\left({\pi}_i\right)=\alpha +{\gamma}_1{G}_{ij}+{\gamma}_2{G}_{ik}+\theta {G}_{ij}{G}_{ik}+{\vartheta}_i+\varepsilon, $$

$$ \vartheta \sim N\left(0,{\sigma}_g^2\Omega \right),\mathrm{and}\ \varepsilon \sim N\left(0,{\sigma}_e^2\right), $$

where *π*_*i*_ = *P*(*Y*_*i*_ = 1| *G*_*ij*_, *G*_*ik*_, *ϑ*) is the probability of disease for subject *i*, conditional on SNPs *G*_*ij*_, *G*_*ik*_ and random effects *ϑ*_*i*_. Here, *ϑ* is a *N* × 1 vector of random effects assumed to follow a multivariate Gaussian distribution, ${\sigma}_g^2$ is the additive genetic variance, and Ω is the genetic similarity matrix between all pairs of individuals (dimension *N* × *N* ) such that Ω_*il*_ represents the similarity between individuals *i* and *l*. An estimate of the genetic similarity matrix, Ω, is required which can be obtained from a large number of genetic variants [33]. Fitting the model involves integrating over the random effects vector *ϑ* with respect to the Gaussian distribution so that the likelihood is maximized with respect to the parameters $\left\{\alpha, {\upgamma}_1,{\upgamma}_2,\uptheta, {\sigma}_g^2,{\sigma}_e^2\right\}$ [34]. In MBMDR-PG we obtain the adjusted phenotype from the residuals of fitting the logistic random effect model using the R package *GMMAT* (Generalized Linear Mixed Model Association Test) [32]. Then, similar to MBMDR-PC we use the adjusted phenotype as input for interaction analysis with MBMDR.

### MBMDR-GC: accounting for genomic structure via genomic control

The genomic control method introduced in [35] is computationally simple and fast to control for population structure in case-control association studies. The key idea is to divide the observed association test statistic by a single factor, *λ*_*GC*_, which measures the overall inflation in the association test statistic due to population stratification. The factor *λ*_*GC*_ can be estimated by dividing the mediums of the observed association test statistics across a set of markers by the theoretical median of the association test statistic. Notably, corrective factors computed in this sense may turn out to be less than 1 and may inflate observed test values rather than deflating them. Although genomic control has proven useful in a variety of contexts, Price et al. [23] pointed out that the common deflation factor applied to all SNPs where some SNPs differ in their allele frequencies across ancestral populations more than others could lead to loss of power. As a solution, [36] considered test specific genomic control. MBMBDR-GC also employs test-specific genomic control, adapted to the MB-MDR testing framework.

**Fig. 1:**
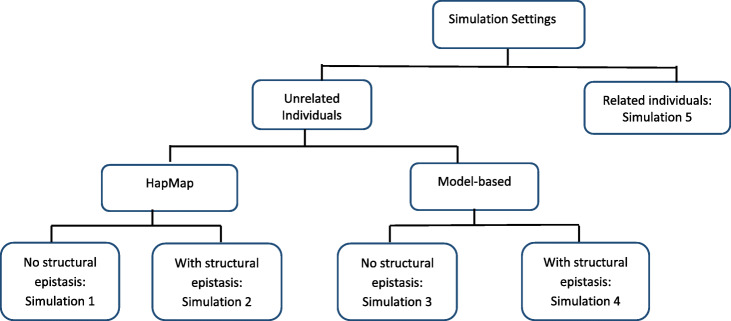
Flow of considered simulation settings

In MBMBDR-GC principles of classic GC in GWAS for structured populations are adopted [23]. Large differences between several multi-locus genotype frequencies across populations may lead to power loss when a single corrective inflation factor GC is used. Therefore, in MBMDR-GC the definition of GC is adapted and the permutation null data generated in MBMDR (*step 3*, Fig. 1) is exploited to estimate a test-specific GC factor, similar to [36]. In particular, for the j^th^ SNP-SNP interaction pair, the corrective factor *λ*_*GC*, *j*_ is estimated as

$$ {\lambda}_{GC,j}=\frac{The\ median\ of\ observed\ interaction\ test\ statistics\ across\  all\  pairwise\ interactions}{The\ expected\ median\ of\ the\  jth\  SNP- SNP\  interaction\ test\ statistic\ {T}_j},\kern0.5em j=1,\dots, J, $$

where *J* is the total number of pairwise interactions and for which the expected median of the j^th^ SNP-SNP interaction test statistic is computed from 1000 permutations under a null distribution constructed by randomly permuting the phenotype values. Then the adjusted test statistic for the j^th^ interaction pair becomes *T*_*j*_/*λ*_*GC*, *j*_, which serve as input to the MB-MDR multiple testing routines instead of their unadjusted counterparts.

### Application on synthetic HapMap data

To assess type I error control and power performance of MBMDR-PC, MBMDR-PG, and MBMDR-GC, and to compare it to MDR-SP [22], we set up a series of simulation settings, involving either unrelated or related individuals, as depicted in Fig. 1. Two main strategies were adopted for our simulation study with unrelated individuals involving: 1) HapMap data as a template with and without structural epistasis, 2) newly generated discrete populations without a reference template, with and without structural epistasis (referred to as model-based data). More detailed explanations are given next.

In summarizing the simulation results, type I error rates were obtained as the proportion of the number of simulated datasets for which a pair of SNPs was found significant at the 5% level after correcting for multiple testing. Similarly, the power was obtained as the proportion of the number of simulated datasets for which only the functional pair of SNPs was found significant at the 5% level after correcting for multiple testing.

#### *Simulation setting 1*: *Synthetic* data derived from HapMap in the absence of structural epistasis

For each simulated set, we considered 200, 300, or 400 individuals, each time equal proportions of cases and controls, and labeled 80% (40%) of controls (cases) as European (CEU) and 20% (60%) of controls (cases) as African (YRI). We followed the simulation strategy adopted for MDR-SP [22] to generate genotype data with unlinked SNPs. In particular, *L* ∈ {200, 400, 800}, independent SNPs were randomly selected from the total number of SNPs from the pooled HapMap3 (CEU and YRI) data with quality control (including only founders, HWE *p*-value threshold of 0.001, individual and genotype missing rates of 5 and 2%, respectively, minor allele frequency *MAF* > 0.05 and LD pruning threshold of *r*^2^ = 0.75) (http://www.sanger.ac.uk/resources/downloads/human/hapmap3.html), and minor allele frequencies were extracted for these two populations. Genotypes were then generated under the assumption of Hardy-Weinberg equilibrium (HWE). The genotypes for the *L* unlinked SNPs were subsequently used to compute principal components and the first 10 principal were retained to capture population substructure.

Since the aim of this study is not to evaluate multiple testing strategies but to evaluate approaches for population structure control in epistasis, SNPs screened for epistasis were generated as follows. A total of only 10 candidate SNPs were selected at random from the available CEU and YRI SNP panels, with the restriction that the minor allele frequency difference between CEU and YRI was larger than *d ϵ* {0.1, 0.3}. Genotypes for unlinked null loci were generated as above. A total of 1000 replicates of null data (i.e., no association between SNPs and trait) were created by repeating the process above 1000 times and by randomly assigning individuals to disease. To be able to assess power, each genetic replicate was appended with 2 functional SNPs. Disease status generation was based on 6 pure epistasis models (Supplementary material – Table S1). These models are heavily used in the epistasis field, for instance, to evaluate MDR [37], MDR-PDT [38], MBMDR [24], and MDR-SP [22]. They involve equal MAFs for functional SNP pairs, with MAFs ∈ {0.50, 0.25, 0.10} and no main effects. We randomly selected 2 SNPs from the pooled CEU and YRI HapMap data ensuring that the MAF in the CEU population at each of the 2 SNP was within ±0*.*02 of the given MAF in the chosen pure epistasis disease model. Two-locus genotypes for the functional SNP pair were then generated, conditional on fixed and equal numbers of cases and controls (100, 150, and 200) each. This process was repeated 1000 times.

#### *Simulation setting 2*: synthetic data derived from HapMap with structural epistasis

To introduce structural epistasis into our simulation study for GWAIS, we considered four HapMap populations: 2 closely related populations CHB and JPT (F_ST_ = 0.007) and 2 distant populations CEU and YRI (F_ST_ = 0.153). Then, to detect epistasis via adjusting nonlinear structural differences between these populations we applied the aforementioned MB-MDR methods for structured populations. We subsequently identified all significant SNP-SNP interaction pairs, adjusted for main effects, and corrected for multiple testing with default options. Based on these results, several approaches were taken to generate genotypes in the absence or presence of epistatic differences between populations. Approach 1: we generated 10,200 unlinked random genotypes including a) 10,000 SNPs randomly generated from the pooled CEU, YRI, CHB, and JPT data, without association to disease and population structure, similar to simulation set 1, and b) 100 pairs of SNPs randomly selected from the aforementioned significant pairs of SNPs related to population structure comparing CHB to JPT, and CEU to YRI. From these 100 pairs, we extracted the empirical proportion of corresponding 9 two-locus genotype combinations. The associated penetrance functions were used to generate the additional 200 unlinked genotypes, by conditioning on fixed sample sizes of {100, 250} from each of the four populations. Approach 2: we simulated 110 candidate random genotypes including a) 100 without association to disease with population structure similar to Approach 1 -b) and 5 significantly interacting SNP pairs with population structure similar to Approach 1 -b). Approach 3: Two functional genotypes were randomly selected from the significant pairs that were found to be associated with population structure in such a way that the MAFs in the CEU and CHB populations at each SNP were within ±0*.*1 of the given MAFs in the disease model (Supplementary Table S1). A total of 1000 replicates were generated for total samples sizes of {400, 1000} and proportions of cases and controls according to 60:40.

Unlinked genotypes obtained via Approach 1 were used to extract principal components to control for population structure. The first 10 principal components were used to capture population structure in epistasis analyses. Candidate genotypes generated via Approach 2 were used to evaluate type I error rates of proposed population correction strategies in GWAIS, whereas functional genotypes as in Approach 3 were used in methods power analyses. Various ways of computing principal components were implemented to capture synthetic data underlying population structure. In particular, we considered linear principal component analysis (linear PCA), as applied to genetic data in [23], kernel PCA with a radial basis kernel, as implemented in the R package *kernlab* (Kernel-Based Machine Learning Lab)*,* and ncMCE (non-centered Minimum Curvilinear Embedding) kernel-based PCA introduced in [27] as an alternative to capture nonlinear genetic differences between populations.

#### *Simulation setting 3*: model-based discrete populations in the absence of structural epistasis

Here, we simulated a large number of biallelic genotype frequencies for each individual in subpopulations, using Balding-Nicholas models [39], similar to [35, 36]. First, an ancestral allele frequency *p*_*a*_ was randomly sampled from the uniform distribution in the interval [0.05, 0.95]. Second, Wright’s coefficient of inbreeding *F*_*ST*_ was specified for the subpopulations *F*_*r*_ ∈ {0.01, 0.03}, *r* = 1, 2. Third, the allele frequency ${p}_{ij}^{(r)}$ of individual *i* for genotype *j* in subpopulation *r* was simulated from a beta distribution with parameters ${p}_a\left(\frac{F_r}{1-{F}_r}\right)$ and $\left(1-{p}_a\right)\left(\frac{F_r}{1-{F}_r}\right),$ *r* = 1, 2, *i* = 1, …, *N* and *j* = 1, …, *M*. Then, genotype values {0, 1, 2} were simulated from a multinomial distribution with probabilities - computed without assuming HWE - by $\left\{\kern0.50em {F}_t\ {p}_{ij}^{(r)}+\left(1-{F}_t\right){{\left({p}_{ij}^{(r)}\right)}^2}_{,}\ 2\left(1-{F}_r\right)\ {p}_{ij}^{(r)}\left(1-{p}_{ij}^{(r)}\right),{\mathrm{F}}_r\left(1-{p}_{ij}^{(r)}\right)+\left(1-{F}_r\right){\left(1-{p}_{ij}^{(r)}\right)}^2,\right\}$ (see [36] and references therein). We thus generated 1000 unlinked SNPs to calculate principal components similar to [23]. Also, 100 SNPs were generated similarly to the unlinked SNPs to evaluate type I error rates. For power comparison, two functional SNPs were generated taking into account the six genetic disease models presented as supplementary information (Table S1). This procedure was repeated 1000 times with total samples sizes of {500, 1000} and proportions of cases and controls according to 60:40.

#### *Simulation setting 4*: model-based discrete populations in the presence of structural epistasis

Instead of relying on a data-driven empirical penetrance table for structural epistasis as before (Simulation setting 2), we considered a checker-board type of model as in Table 1, which describes epistatic genetic differences between the populations using the XOR model. In Table 1, the parameter *β*_0_ was taken to be the average penetrance (in the absence of any genetic effect), whereas *β*_1_ captured the increase in penetrance when having the specific 2-locus genotype. In our simulations we assumed *β*_0_ = 0 and *β*_1_ = 0.35 and 0.20 for populations 1 and 2, respectively. Then, we generated 1000 unlinked random genotypes including a) 800 SNPs randomly generated similar to *Simulation setting 3* using *F*_*ST*_ in the two subpopulations *F*_*r*_ ∈ {0.001, 0.001}, *r* = 1, 2; b) 100 pairs from each population similar to *Simulation setting 2 (1b)* using the population-specific penetrance values given in Table 3 with *β*_*j*, 1_ = *β*_1_ + *ε*, *j* = 1, …, 50, where *ε* is randomly drawn from *uniform* (0, 0.05). To assess type I error rate, 120 SNPs are generated of which 100 similar to (a) and 10 pairs similar to (b). A total of 1000 replicates were generated for total samples sizes of {200, 500, 1000} and proportions of cases and controls according to two scenarios 60:40 and 80:20. This dataset was used to construct principal components.

**Table 1:**
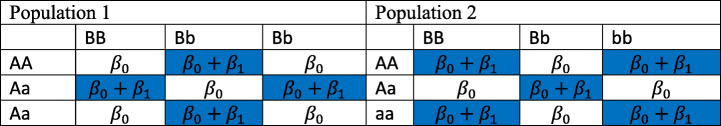
Checkerboard stratification penetrance models for structural epistasis

#### *Simulation setting 5*: simulating genotypes for related individuals

Inspired by [40], we simulated 1000 replicate datasets consisting of 250 nuclear families, with the number of children drawn from a multinomial distribution with probabilities 1/4 to have one child, 1/2 to have two children, and 1/4 to have three children. On average, this gave rise to 1000 individuals. To generate parental genotypes, we generated 10 biallelic markers in linkage equilibrium and assuming Hardy-Weinberg equilibrium. The allele frequencies of the functional SNP pair (*SNP*_1_, *SNP*_2_) were taken to be equal, and varied as (*p*_1_, *p*_2_) = (*p*, *p*), *p* ∈ (0.1,0.25,0.5) . The allele frequencies of the 8 remaining non-functional SNPs were fixed at *p*_*j*_ = 0.1 + (*j* − 3)0.05, *j* = 3, …10. Children’s genotypes were assumed to follow Mendelian inheritance patterns. Disease penetrance for parents and children was based on Model M170, as discussed in [41]. This epistasis model is similar to Model 1 in Table S1 (supplementary material). However, we fixed the total heritability *h*^2^ and the proportion of the total variance explained by the two-locus model variance at 0.5 and 0.05, respectively. As family relationships may induce phenotype similarity, this simulation setting was used to evaluate the performance of MBMDR-PG.

## Results

### Simulation setting 1

Type I error estimates obtained for simulation setting 1 via application of MBMDR-PC, MBMDR-PG, MBMDR-GC, and MDR-SP to 1000 replicated samples are presented in Table 2. In the case of a single homogeneous population (CEU only) none of the estimated type I errors is significantly different from the nominal 0.05 FWER level, with a 95% confidence interval of (0.036, 0.064) [22]. This is the case, for all considered combinations of population structure correction methods, sample sizes, and number of SNPs. In the case of structured samples (in particular, consisting of CEU and YRI), MBMDR-PC estimated type I errors presented in Table 2 always follow Bradley’s liberal criterion. In addition, it can be seen from Table 2 that all the estimated type I error rates for MBMDR-PC are within the 95% confidence interval but it is not the case for MDR-SP. However, many type I error rate estimates based on MBMDR-GC do not fall within the 95% interval. The results of MBMDR-PG are similar to MBMDR-PC (results not shown).

**Table 2:** Estimates of Type I error for MBMDR-PC, MBMDR-GC, and MDR-SP, with a nominal 0.05 FWER level

			*b* = 40%	
Method	Sample sizes	Markers	*d* = 0.1	*d* = 0.3
MBMDR-PC	200	200	0.050	0.046
		400	0.051	0.051
		800	0.046	0.048
	300	200	0.046	0.047
		400	0.047	0.049
		800	0.049	0.047
	400	200	0.050	0.053
		400	0.051	0.054
		800	0.048	0.046
MDR-SP	200	200	0.054	0.054
		400	0.062	0.055
		800	0.062	0.050
	300	200	0.051	0.056
		400	0.055	0.051
		800	0.044	0.046
	400	200	0.044	0.050
		400	0.046	0.052
		800	0.044	0.065
MBMDR-GC	200	200	0.059	0.058
		400	0.061	0.065
		800	0.064	0.067
	300	200	0.060	0.065
		400	0.062	0.071
		800	0.063	0.068
	400	200	0.066	0.069
		400	0.061	0.071
		800	0.063	0.066

Note: *d* denotes the difference of candidate allele frequencies in the two subpopulations, and *b* denotes the percentage of cases from the European subpopulation

**Fig. 2:**
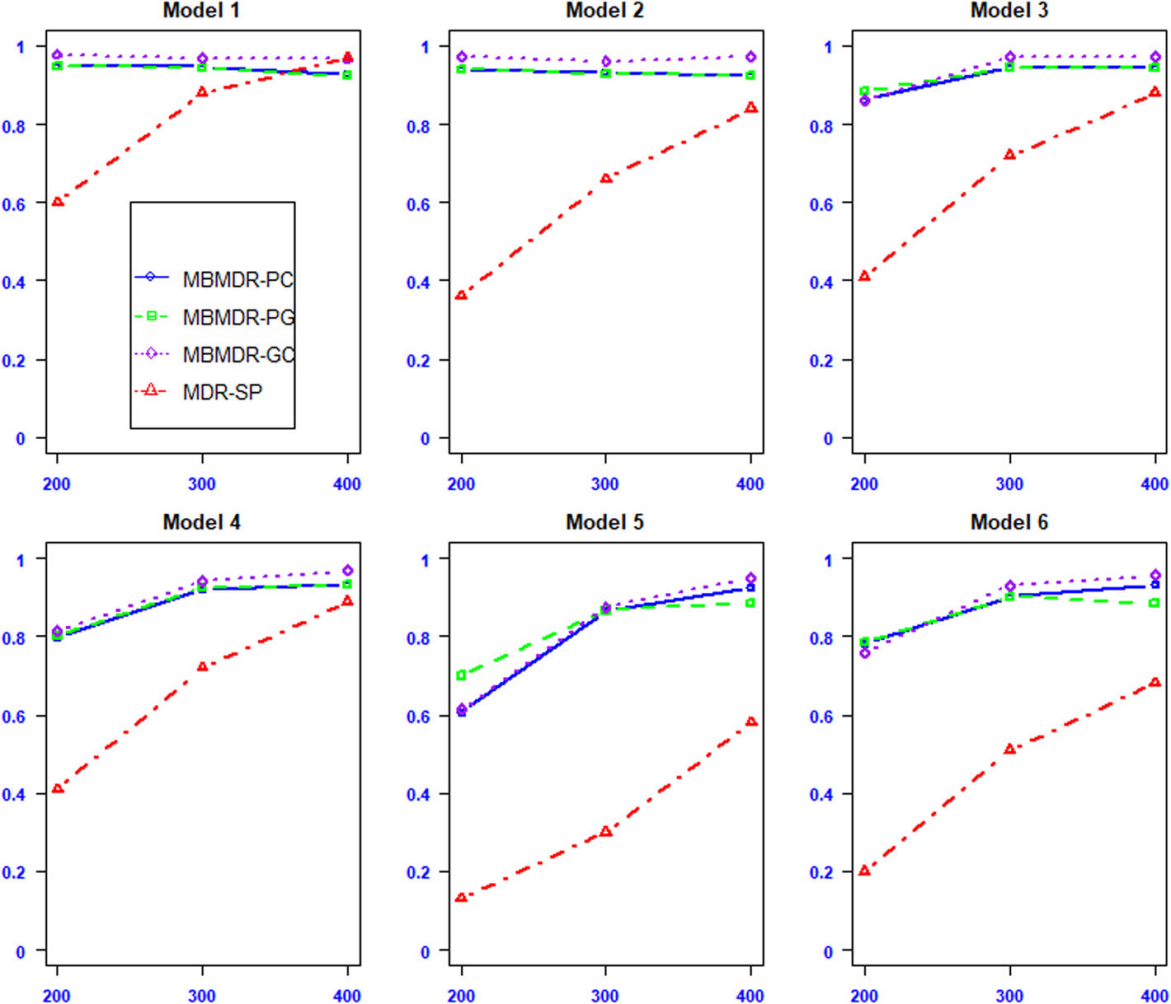
Power estimates for MBMDR-PC (blue/solid line), MBMDR-PG (green/dashed line) and MDR-SP (red/dotted line) under the six disease models based on simulated data on CEU and YRI populations with a difference of 0.3 minor allele frequency between the two populations. Percentage of cases and control from the CEU are 40 and 80%, respectively. The power (y-axis) is computed using 10 candidate SNPs. PCs are computed from 200

In Fig. 2, for allele frequencies difference *d* = 0.1 and European population percentage *b* = 40%, MBMDR-PC is significantly more powerful than MDR-SP under all models considered in particular for small sample sizes. Moreover, MBMDR-PC outperforms MDR-SP even for large sample sizes in models 5 and 6. Notably, these epistasis models are the toughest of the 6 considered Ritchie models in that they involve functional SNP pairs with the lowest MAFs (0.10). As the sample size increases the power of both methods increases. We also included the power results of MBMDR-PG, which are almost similar to MBMDR-PC. Similar results follow when *d* = 0.3 and *b* = 40%. In addition, the results of power based on varying number of unlinked markers are included in Fig. S1 (Supplementary material) that suggest there is not much difference in the power of MBMDR-PC using 200, 400, and 800 unlinked markers for computing principal components to control population structure in our data simulation.

### Simulation setting 2

**Fig. 3:**
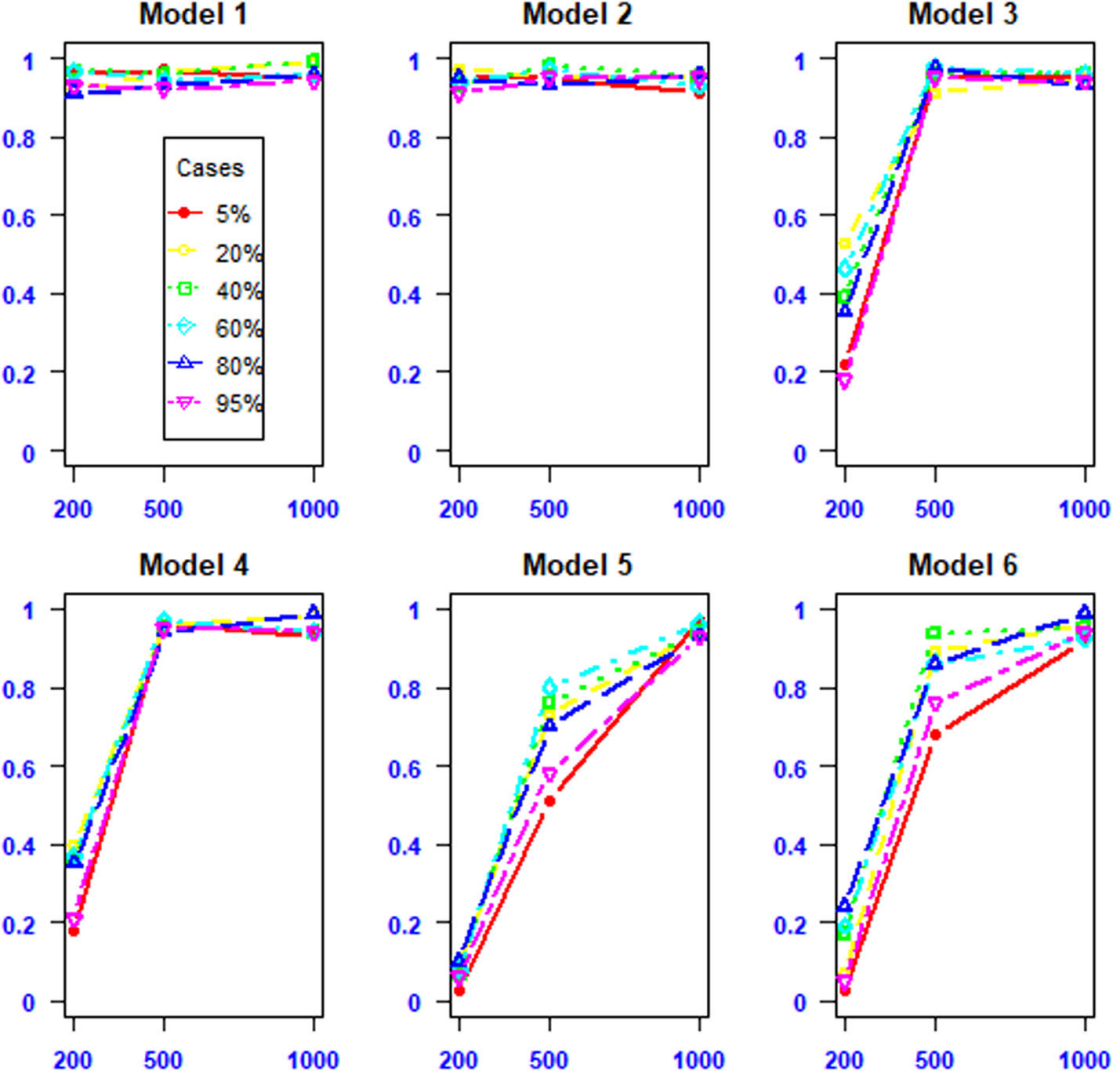
Power estimates according to varying proportions of cases and controls in six disease epistasis models and variable sample sizes (200, 500, 1000). The percentage of cases in one of the two populations are shown

The estimated power of MBMDR-PC under the discrete population simulation setting is shown in Fig. 3. The results show that MBMDR-PC has high power in all scenarios of varying case-control proportions in all disease models with large samples. The power of MBMDR-PC is low for small sample sizes (100 samples from each of the two populations) for disease models with moderate and small minor allele frequencies. In general, the simulation results of varying case-control proportions have no considerable impact on the power of MBMDR-PC method for large samples of 1000 or more. The results of estimated Type I error rates for varying proportions of cases and controls with and without main effect and principal component corrections are displayed in Fig. S2 (Supplementary material). From this figure we see that MBMDR-PC performs well in controlling type I error rate at the nominal 0.05 FWER level with and without main effects correction in all scenarios of case-control proportions (Fig. S2 A and B). Use of the original MBMDR without population and main effect corrections in case of structured population leads to inflated type I error rates (Fig. S2 D and C) in case of small samples and a large difference in case-control proportions.

### Simulation setting 3

**Fig. 4:**
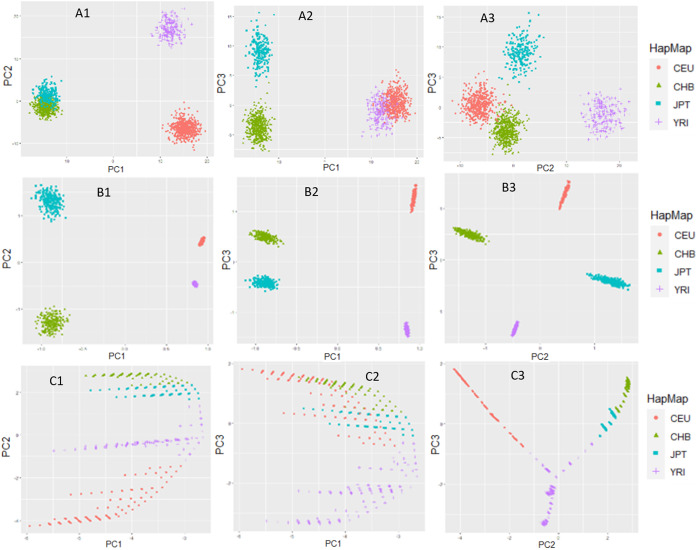
Pairwise plots of the first three principal components computed using linear (A1-A3), kernel (B1-B3) and ncMCE (C1-C3) PCA methods

To evaluate the performance of MBMDR-PC in multiple subpopulations we evaluate three principal component extraction methods: linear, kernel, and ncMCE. Pairwise PC-plots for the first three principal components computed from the unlinked null SNPs are shown in Fig. 4. The plot of the first and the second PCs obtained from linear PCA (Fig. 4 A1) fails to separate CHB and JPT populations. Similar results were reported in [27]. However, the plot of the second and third linear PCs (Fig. 4 A3) differentiate all four populations. In the case of kernel-based PCs, the four populations are separable in any of the pairwise PC plots of the first three kernel PCs (Fig. 4 B1-B3). On the other hand, the plot of the first versus the second ncMCE based PCs (Fig. 4 C1) was able to reveal the hierarchical structure of the four populations, reflecting the phylogenetics of these populations, as discussed in Alanis-Lobato and colleagues [27].

As can be seen from Table 3, none of the considered simulation scenarios show marked differences regarding type I error control or power to detect epistasis, when using the first 10 PCs computed via linear, kernel, or ncMCE PCA methods with MBMDR-PC. In contrast, type I error estimates are somewhat inflated with MBMDR-GC. However, MBMDR-PC and MBMDR-GC give comparable power estimates, except for epistasis models with low-frequency causal variants (Models 5 and 6).

**Table 3:** Estimates of power and type I error rates of MBMDR-PC with population structure captured by linear, kernel, and ncMCE principal components and MBMDR-GC, with a nominal 0.05 FWER level

	MBMDR-GC		MBMDR-PC					
			Linear PCA		Kernel PCA		ncMCE	
Sample sizes	200	500	200	500	200	500	200	500
Type I								
Error Rates	0.077	0.085	0.052	0.054	0.054	0.050	0.047	0.055
Power								
Model 1	0.945	0.735^a^	0.927	0.730^a^	1.000	0.740^a^	0.929	0.770^a^
Model 2	0.805	0.131^a^	0.846	0.404^a^	0.864	0.560^a^	0.895	0.556^a^
Model 3	0.653	0.954	0.658	1.000	0.731	0.970	0.669	0.968
Model 4	0.483	0.956	0.481	0.960	0.476	0.945	0.490	0.960
Model 5	0.074	0.784	0.259	0.950	0.238	0.950	0.258	0.958
Model 6	0.161	0.918	0.447	0.970	0.421	0.970	0.446	0.956

^a^The values are close to 1 when the power is calculated based on all significant interactions that include the true signal

### Simulation setting 4

**Fig. 5:**
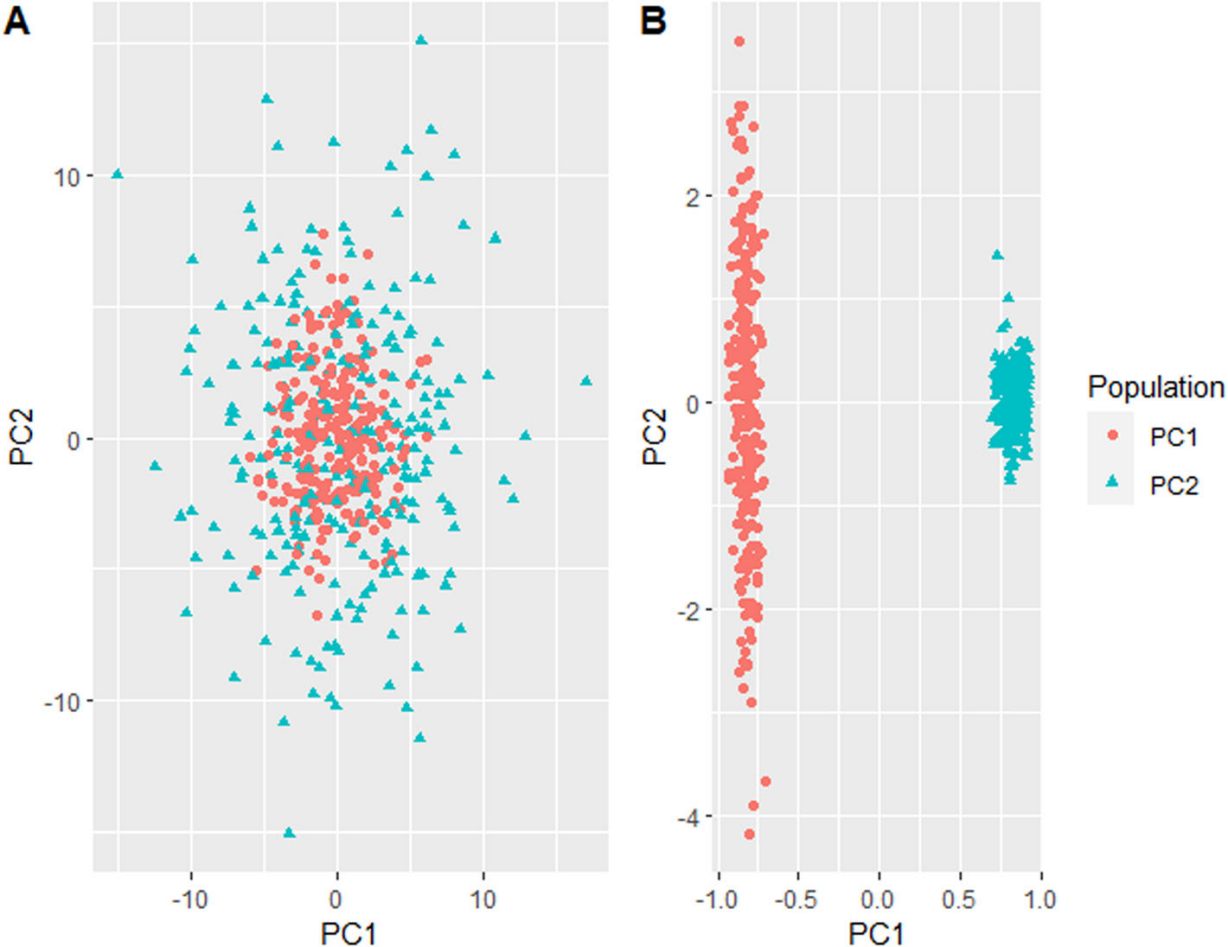
Plots of the first two principal components (**a**) linear PCA and (**b**) kernel PCA

**Fig. 6:**
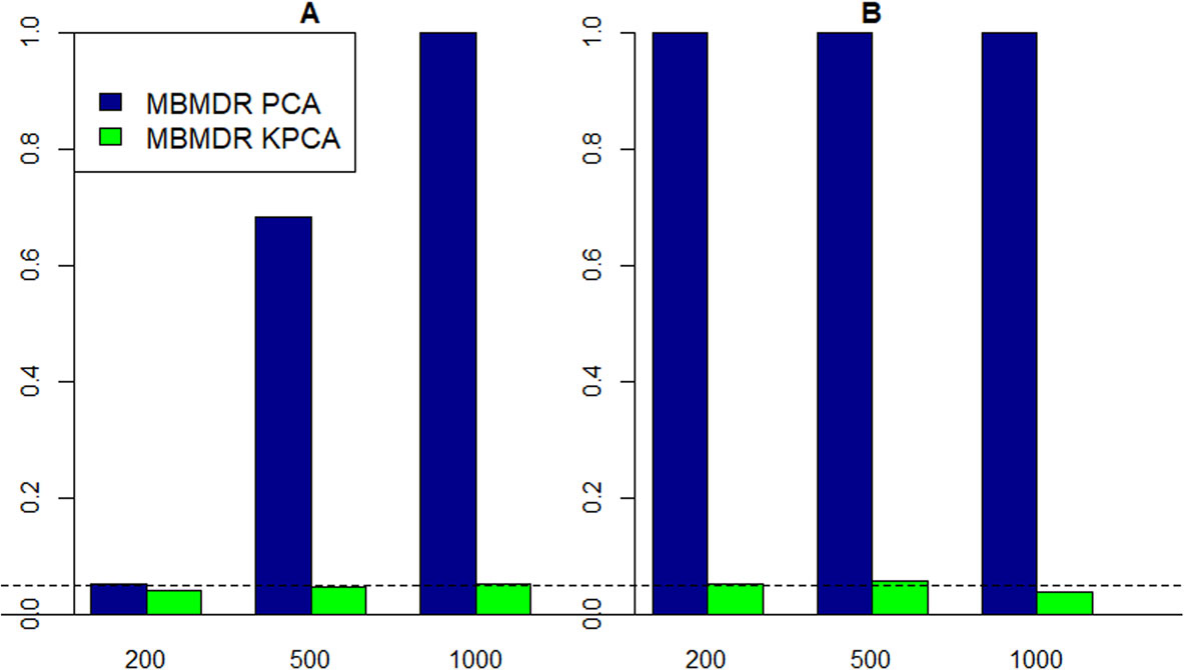
Estimated type I error rates for MBMDR-PC with case-control ratios (**a**) 60:40 and (**b**) 80:20. PC approaches considered: linear PCA (blue bars), kernel PCA (green bars)

The scatter plot on the first 2 linear and kernel principal components for a single simulated dataset (see Methods section) is shown in Fig. 6. Linear PCA indicates a nonlinear genetic background structure (Fig. 5a). This is confirmed by kernel-based PCA, which separates the two subpopulations (Fig. 5b).

The estimated results of type I error rates of MBMDR-PC using linear and kernel principal components are presented in Fig. 6. In the presence of phenotypic and structural epistasis, linear PCA-based MBMDR-PC highly inflates the type I error which is substantially higher than the nominal 0.05 FWER level. For example, for a total sample size of 500 (cases and controls jointly) and case-control ratios 60:40 and 80:20, the type I error rates of linear MBMDR-PC, at a nominal level of 0.05, are 0.7 and 1.0, respectively (Fig. 6a and b). Type I error rates of linear MBMDR-PC increase as the sample size increases. Furthermore, type I errors estimates get worse for linear PCA based MBMDR-PC with increasing levels of unbalancedness (Fig. 6b, 80:20). In comparison, the estimated type I error rates of kernel-based MBMDR-PC are not significantly different from the nominal level 0.05 in all the scenarios considered.

### Simulation setting 5

The estimated type I error rate for simulation setting based on related samples obtained from 1000 replicates (as explained in the Methods section) is 0.051, which is close to the nominal 0.05 level. Power estimates for epistasis model M170 (see Methods) increase with increasing minor allele frequencies for the causal epistasis SNP pair (0.45, 0.885, and 0.911 for MAFs of 0.1, 0.25, and 0.5, respectively).

## Discussion

In this work, we have highlighted the importance of detecting and correcting for population structure in epistasis studies. Using extensive simulations we have shown that in genome-wide epistasis studies inappropriate correction for complex population structure results in inflated false positives or low power to detect true signals of epistasis. When evaluating the impact of ignored or inadequately captured population structure in GWAIS, we not only considered epistatic ancestry informative markers but also paid special attention to the idea of nonlinearity in population genetics [27]. Additionally, we considered the influence of unequal sample sizes. As epistasis detection analysis tool, we relied on MB-MDR, which should be seen as part of an entire analysis pipeline that involves making marker selection choices and performing post-analysis steps to validate and replicate findings, as well as seeking biological evidence for flagged interacting regions [18].

That ignoring population structure due to allele frequency differences among populations and subpopulations can result in high numbers of false positives or reduced power in GWAS is not new. In GWAS, Structured Association (SA) [42–45], Genomic Control (GC) [35, 36], Principal Component Analysis (PCA) [23] and Mixed Modeling (MM) [46] are the main 4 strategies to deal with confounding associations due to shared genetic ancestry. The basic idea of SA is to infer the underlying population structure and then to incorporate this information in subsequent testing for genetic associations of interest. In contrast, the basic idea of GC is to correct the null distribution of genetic association tests for the effects of the unspecified population structure [35]. The statistical advantage of SA methods depends on the degree of information provided by the available marker data to make inferences about the true structure. Classic GC methods rely on adjusting all marker-trait associations in the same way, which ignores the strength of the relationship between the genealogy of the genetic marker under study and the (hidden) pedigree structure, and thus also dependencies between markers. The basic idea of MM for controlling population structure is to account for pairwise relatedness between individuals, for example, using a kinship matrix. It is an approach that naturally accommodates familial and cryptic relatedness in the data. Mixed models have long been computationally expensive; it took until the development of more efficient algorithms for them to gain popularity in population structure control. Some of the algorithm improvements are incorporated in the following approaches: compressed-MLM [46], EMMA (Efficient Mixed-Model Association) [47], EMMAX (EMMA eXpedited) [48], GEMMA (Genome-Wide Efficient Mixed-Model Association) [49], LRLMM (low rank linear mixed model [33], FaST-LMM (Factored Spectrally Transformed Linear Mixed Model) [50], FaST-LMM-Set [51], GRAMMAR-Gamma (fast variance components-based two-step method) [52], and FarmCPU (Fixed and random model Circulating Probability Unification) [53]. PCA allows data transformation to a new coordinate system such that the projection of the data along the first new coordinate has the largest variance, the second principal component has the second largest variance, and so on. The relative straightforwardness of PCA, its ease of use, the availability of efficient algorithms, and its ability to detect individuals with unusual or differential ancestry [28, 54–58] has made PCA among the most heavily used strategies in the context of genetic association studies in structured populations. Once principal components are obtained, several choices can be made to use these for the purpose of confounding correction in GWAS. Assuming that the GWAS is performed within a regression framework, the most straightforward approach is to include the first few principal components, capturing genetic ancestry of each individual, as fixed effects in a (generalized) linear model. Alternatively, instead of directly including the principal components in a regression model, both phenotype and genotypes can be adjusted by top PCs as in EIGENSTRAT [23]. The adjusted phenotype is defined as the residual of fitting an appropriate generalized linear regression model of phenotype on a number of principal components. A similar model fitting is performed to obtain adjusted genotypes [12].

The aforementioned methods naturally extend to epistasis detection frameworks, in particular those that allow for a regression model component in their methodology. One such framework is MB-MDR (32), which adds a model-based component to Multifactor Dimensionality Reduction, hereby enabling adjusting for lower-order genetic effects or confounders (46). Our proposed methods for detecting epistasis in the presence of population structure, MBMDR-PC, MBMDR-PG, and MBMDR-GC, build on MB-MDR. MBMDR-PC and MBMDR-PG involve first deriving adjusted phenotypes (residuals) obtained from fitting appropriate generalized linear models with the first few principal components (linear or nonlinear) as covariates, and generalized linear mixed models with a kinship matrix to capture the covariance structure of random effects, respectively. MBMDR-GC involves computing SNP-pair specific correction factors for each MB-MDR observed test value. This was inspired by earlier observations that the distribution of MB-MDR test statistics may largely vary from one SNP-pair to another due to a combination of disease prevalence and minor allele frequencies of SNPs under testing (results not shown). The generated null data under the hypothesis of no trait associations are used twice with MBMDR-GC: first to estimate the expected MB-MDR test value for each SNP pair *j* under this null, and second to assess the statistical significance of observed MB-MDR test values that are adjusted by *λ*_*GC*, *j*_. With equal MB-MDR test null distributions across SNP pairs, no genetic associations with the trait and no population structure, the expected *λ*_*GC*, *j*_ should approximate 1. With unequal MB-MDR test null distributions, observed test values will receive higher chances to become significant with higher expected SNP-pair related median test values, computed in the absence of genetic and confounder associations with the trait.

In general, our simulation results showed that in the presence of population structure MBMDR-PC and MBMDR-PG consistently control type I error rate at the nominal level compared to MBMDR-GC which had a slightly inflated type I error rate. Also, our three methods of population structure correction were more powerful than MDR-SP. Thus, MBMDR-PC and MBMDR-PG for GWAIS adjusted for confounding by (non) linear population structure give promising results and are to be preferred over MDR-SP in the considered simulation settings. Our results also suggested that there is no need to compute population controlling PCs for every SNP pair separately. For related samples, MBMDR-PG based on a generalized linear mixed model should be used. In many instances of mild population structure, MB-MDR with codominant correction exhibits comparable performance to MBMDR-PC. All analyses can easily accommodate covariates using similar principles as in MBMBDR-PC and MBMDR-GC.

Epistasis studies may benefit from consortium-based sample collections, where large sample sizes can boost the power of epistasis detection. For instance, the International Inflammatory Bowel Disease Genetics Consortium comprises data from 68,427 samples in 15 countries [59]. However, large sample sizes may also increase heterogeneity and possible interferences of population structure. To investigate how type I error and power were affected by structured populations with thousands of samples, we repeated simulation setting 1 (percentage of cases and control from CEU being 40 and 80%, respectively, and differences in minor allele frequencies of candidate SNPs in CEU and YRI being d = 0.3), this time with 10,000 (instead of maximum 400) samples. Restricting to MBMDR-PC, type I error remained controlled at 0.05; Power was estimated as 100% for Models 1 through 6. Notably, MB-MDR was shown before to scale with increasing number of samples [10]; however, alternative computation-time efficient algorithms may be required to compute the principal components needed for capturing population structure in large samples. Here, we used the R package Rspectra. Alternative packages in R include fastpca, flashpca, or bigpca.

The outperformance of MBMDR-PC depends on the ability of the principal components to capture the population structure well. We chose the checkerboard stratification model to inject strong nonlinear genetic differences between two populations; more work is needed to investigate a variety of complex nonlinear stratification models and to assess their occurrence in real-life. Overall, widely used linear PCA fails to properly differentiate such complex populations Kernel-based strategies offer an interesting alternative, especially when additional efficient computational tools are developed to extract non-linear PCs from large genetic datasets as those collected within disease-specific consortiums. Our simulation results that compare MBMDR-PC with linear and kernel PCs showed that MBMDR-PC with linear PCs gives inflated type I errors, which becomes worse as the ratio of case-control becomes increasingly unbalanced. On the contrary, MBMDR-PC based on kernel PCs effectively controlled for both linear and non-linear population structure and maintained the type I error rates at the required nominal levels.

In conclusion, MBMDR-PC is a generally well-performing approach, compared to the computationally intensive MBMDR-PG and MBMDR-GC approaches, although its performance is highly dependent on how well PCs capture population structure. Therefore, we recommend using both linear and nonlinear versions of PCA, whenever possible. Fast implementation for multiple testing correction in exhaustive epistasis screenings [10] makes our proposed MB-MDR based methods efficient tools for GWAIS in structured populations. Our work is important given ongoing initiatives of epistasis detection in large-scale heterogeneous consortium data, as we have shown that inadequate capturing of population structure may severely jeopardize obtaining meaningful and replicable epistasis findings.

## Supplementary Information

**Additional file 1: Table S1.** Pure epistasis disease models used in the simulation to evaluate the power of MBMDR methods for structured populations. **Fig. S0.** Power comparisons of MBMDR-PC (blue solid line) and MDR-SP (red dashed line) under the six disease models based on simulated CEU population. The power (y-axis) is computed using 10 candidate SNPs and 200 unlinked SNPs used to compute principal components with varying sample sizes (x-axis). **Fig. S1.**. Power comparisons of MBMDR-PC of a varying number of SNPs in PC computation under the six disease models based on simulated data on CEU and YRI populations with a difference of minor allele frequencies of candidate SNPs greater than 0.3 between the two populations and percentage of cases and control from the CEU are 40 and 80%, respectively. The power (y-axis) is computed using 10 candidate SNPs and 200 (red), 400 (green), and 800 (blue) unlinked SNPs to control population structure via principal components with varying sample sizes (x-axis). **Fig. S2.** Results of type I error according to varying proportions of cases and controls. The percentage of cases in one of the two populations are shown. MBMDR methodology with (A) PCs and main effects correction, (B) only PCs correction, (C) only main effects correction, and (D) no correction.

## Data Availability

Codes to implement MBMDR-PC, MBMDR-PG and MBMDR-GC are available via the MBMDR software (from version mbmdr-4.4.1 onwards), which is downloadable from http://bio3.giga.ulg.ac.be/index.php/software/mb-mdr/ . The following include the main options used in this study. MBMDR-PC: *--binary –ac number of PCs –d 2D –a CODOMINANT –rc RESIDUALS.* MBMDR_PG: *--continuous*^***^ *–d 2D –a CODOMINANT.* MMBMDR_GC: *--binary –d 2D –mt STRAT3.* **: residuals obtained using the R package GMMAT.* Simulation code is available upon request via kristel.vansteen@uliege.be

## Abbreviations

GC: Genomic Control
GWAS: Genome-wide Association Study
GWAIS: Genome-wide Association Interaction Studies
MAF: Minor Allele Frequency
MB-MDR: Model-Based Multifactor Dimensionality Reduction
MDR: Multifactor Dimensionality Reduction
MDR-SP: Multifactor Dimensionality Reduction for Stratified Populations
PC: Principal Components
PCA: Principal Component Analysis
PG: Polygenic
SA: Structured Association
SNP: Single Nucleotide Polymorphism
