# Supplementary material for: Performance of model-based multifactor dimensionality reduction methods for epistasis detection by controlling population structure
Source: BioData Min. 2021 Feb 19;14:16. doi: 10.1186/s13040-021-00247-w (PMC7893746; doi:10.1186/s13040-021-00247-w)
Supplement: Supplementary file 1 — Additional file 1: Table S1. Pure epistasis disease models used in the simulation to evaluate the power of MBMDR methods for structured populations. Fig. S0. Power comparisons of MBMDR-PC (blue solid line) and MDR-SP (red dashed line) under the six disease models based on simulated CEU population. The power (y-axis) is computed using 10 candidate SNPs and 200 unlinked SNPs used to compute principal components with varying sample sizes (x-axis). Fig. S1.. Power comparisons of MBMDR-PC of a varying number of SNPs in PC computation under the six disease models based on simulated data on CEU and YRI populations with a difference of minor allele frequencies of candidate SNPs greater than 0.3 between the two populations and percentage of cases and control from the CEU are 40 and 80%, respectively. The power (y-axis) is computed using 10 candidate SNPs and 200 (red), 400 (green), and 800 (blue) unlinked SNPs to control population structure via principal components with varying sample sizes (x-axis). Fig. S2. Results of type I error according to varying proportions of cases and controls. The percentage of cases in one of the two populations are shown. MBMDR methodology with (A) PCs and main effects correction, (B) only PCs correction, (C) only main effects correction, and (D) no correction. [file 13040_2021_247_MOESM1_ESM.docx]

**Supplementary Material to Performance of Model-Based Multifactor Dimensionality Reduction Methods for Epistasis Detection by Controlling Population Structure**

Fentaw Abegaz^1,^*, François Van Lishout^1^, Jestinah M Mahachie John^1^, Kridsadakorn Chiachoompu^1^, Archana Bhardwaj^1^, Diane Duroux^1^, Elena S Gusareva^1^, Zhi Wei^2^, Hakon Hakonarson^3,4^, and Kristel Van Steen^1,5^

^1^ GIGA-R, Medical Genomics – BIO3, University of Liege, Liege, Belgium

^2^ Department of Computer Science, New Jersey Institute of Technology, Newark, NJ, USA

^3^ Center for Applied Genomics, The Children’s Hospital of Philadelphia, Philadelphia, PA, USA

^4^ Division of Human Genetics, Department of Pediatrics, The Perelman School of Medicine, University of Pennsylvania, Philadelphia, PA, USA

^5^ WELBIO (Walloon Excellence in Lifesciences and Biotechnology), University of Liege, Liege, Belgium * To whom correspondence should be addressed. Tel: +31 621331831; Email: [fentawabegaz@](mailto:fentawabegaz@)yahoo.com

**Method and Materials**

Table S1: Pure epistasis disease models used in the simulation to evaluate the power of MBMDR methods for structured populations

| Model 1, $p=0.5$   \|  \| BB \| Bb \| bb \| \| --- \| --- \| --- \| --- \| \| AA \| 0 \| 0.1 \| 0 \| \| Aa \| 0.1 \| 0 \| 0.1 \| \| aa \| 0 \| 0.1 \| 0 \| | Model 3, $p=0.25$   \|  \| BB \| Bb \| bb \| \| --- \| --- \| --- \| --- \| \| AA \| 0.08 \| 0.07 \| 0.05 \| \| Aa \| 0.1 \| 0 \| 0.1 \| \| aa \| 0.03 \| 0.1 \| 0.04 \| | Model 5, $p=0.1$   \|  \| BB \| Bb \| bb \| \| --- \| --- \| --- \| --- \| \| AA \| 0.07 \| 0.05 \| 0.02 \| \| Aa \| 0.05 \| 0.09 \| 0.01 \| \| aa \| 0.02 \| 0.01 \| 0.03 \| |
| --- | --- | --- | --- | --- | --- | --- | --- | --- | --- | --- | --- | --- | --- | --- | --- | --- | --- | --- | --- | --- | --- | --- | --- | --- | --- | --- | --- | --- | --- | --- | --- | --- | --- | --- | --- | --- | --- | --- | --- | --- | --- | --- | --- | --- | --- | --- | --- | --- | --- | --- |
| Model 2, $p=0.5$   \|  \| BB \| Bb \| bb \| \| --- \| --- \| --- \| --- \| \| AA \| 0 \| 0 \| 0.1 \| \| Aa \| 0 \| 0.05 \| 0 \| \| aa \| 0.1 \| 0 \| 0 \| | Model 4, $p=0.25$   \|  \| BB \| Bb \| bb \| \| --- \| --- \| --- \| --- \| \| AA \| 0 \| 0.01 \| 0.09 \| \| Aa \| 0.04 \| 0.01 \| 0.08 \| \| aa \| 0.07 \| 0.09 \| 0.03 \| | Model 6, $p=0.1$   \|  \| BB \| Bb \| Bb \| \| --- \| --- \| --- \| --- \| \| AA \| 0.09 \| 0.001 \| 0.02 \| \| Aa \| 0.08 \| 0.07 \| 0.005 \| \| aa \| 0.003 \| 0.007 \| 0.02 \| |

**Results**

**One homogeneous population (CEU)**

Here, we compare the power of MBMDR-PC, MBMDR-GC, and MDR-SP under six epistasis models by using varying sample sizes based on synthetic data obtained from one CEU population. The results of power comparisons on this dataset are displayed in Figure S0. The results show that MBMDR-SP and MBMDR-GC were substantially more powerful than MDR-SP in particular when the sample size is small (200) and for models 5 and 6 that determined by small minor allele frequencies.

**
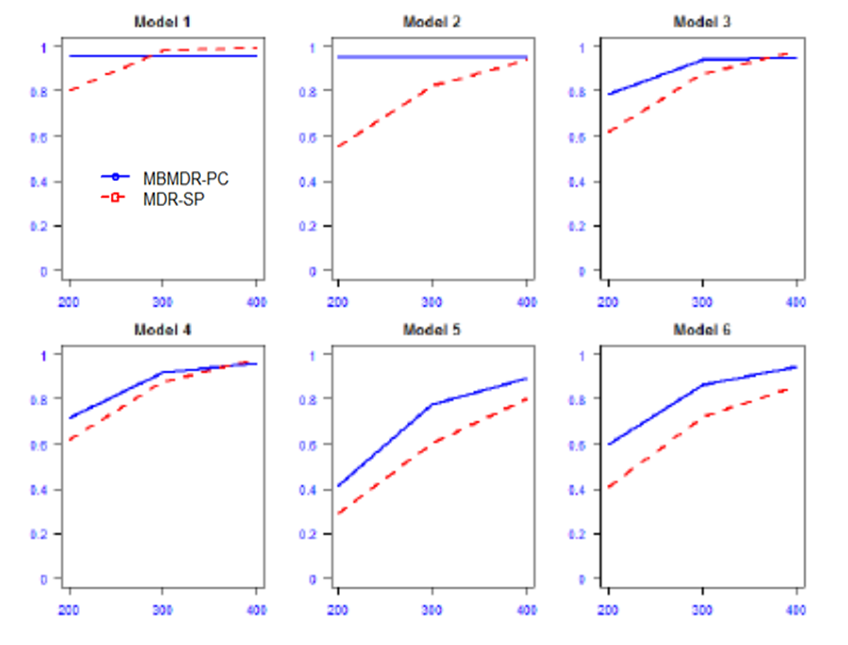
**

Figure S0. Power comparisons of MBMDR-PC (blue solid line) and MDR-SP (red dashed line) under the six disease models based on simulated CEU population. The power (y-axis) is computed using 10 candidate SNPs and 200 unlinked SNPs used to compute principal components with varying sample sizes (x-axis).

Figure S1. Power comparisons of MBMDR-PC of a varying number of SNPs in PC computation under the six disease models based on simulated data on CEU and YRI populations with a difference of minor allele frequencies of candidate SNPs greater than 0.3 between the two populations and percentage of cases and control from the CEU are 40% and 80%, respectively. The power (y-axis) is computed using 10 candidate SNPs and 200 (red), 400 (green), and 800 (blue) unlinked SNPs to control population structure via principal components with varying sample sizes (x-axis).

Sample sizes

Type I error rates

Figure S2. Results of type I error according to varying proportions of cases and controls. The percentage of cases in one of the two populations are shown. MBMDR methodology with (A) PCs and main effects correction, (B) only PCs correction, (C) only main effects correction, and (D) no correction.
